# Inulin Improves Diet-Induced Hepatic Steatosis and Increases Intestinal *Akkermansia* Genus Level

**DOI:** 10.3390/ijms23020991

**Published:** 2022-01-17

**Authors:** Carlos Pérez-Monter, Alejandro Álvarez-Arce, Natalia Nuño-Lambarri, Ivonne Escalona-Nández, Eva Juárez-Hernández, Norberto C. Chávez-Tapia, Misael Uribe, Varenka J. Barbero-Becerra

**Affiliations:** 1 Departamento de Gastroenterología, Instituto Nacional de Ciencias Médicas y Nutrición Salvador Zubirán, Mexico City 14080, Mexico; livon_12ily@hotmail.com; 2 Departamento de Neuropatología Molecular, División de Neurociencias, Instituto de Fisiología Celular, UNAM, Mexico City 04510, Mexico; alejandroarcce@gmail.com; 3 Unidad de Investigación Traslacional, Fundación Clínica Médica Sur, Mexico City 14050, Mexico; nlambarri@gmail.com (N.N.-L.); ejuarezh@medicasur.org.mx (E.J.-H.); khavez@gmail.com (N.C.C.-T.); muribe@medicasur.org.mx (M.U.)

**Keywords:** hepatic steatosis, intestinal microbiota, prebiotics, inulin, *A. muciniphila*

## Abstract

Hepatic steatosis is characterized by triglyceride accumulation within hepatocytes in response to a high calorie intake, and it may be related to intestinal microbiota disturbances. The prebiotic inulin is a naturally occurring polysaccharide with a high dietary fiber content. Here, we evaluate the effect of inulin on the intestinal microbiota in a non-alcoholic fatty liver disease model. Mice exposed to a standard rodent diet or a fat-enriched diet, were supplemented or not, with inulin. Liver histology was evaluated with oil red O and H&E staining and the intestinal microbiota was determined in mice fecal samples by 16S rRNA sequencing. Inulin treatment effectively prevents liver steatosis in the fat-enriched diet group. We also observed that inulin re-shaped the intestinal microbiota at the *phylum* level, were *Verrucomicrobia* genus significantly increased in the fat-diet group; specifically, we observed that *Akkermansia* *muciniphila* increased by 5-fold with inulin supplementation. The family *Prevotellaceae* was also significantly increased in the fat-diet group. Overall, we propose that inulin supplementation in liver steatosis-affected animals, promotes a remodeling in the intestinal microbiota composition, which might regulate lipid metabolism, thus contributing to tackling liver steatosis.

## 1. Introduction

Non-alcoholic fatty liver disease (NAFLD) is the hepatic manifestation of metabolic syndrome. It was proposed that overweight and obesity are closely related to NAFLD both of which are also related to glucose intolerance and insulin resistance. Metabolic syndrome is the result of excessive calorie intake and a corresponding lack of energy expenditure, by physical activity or by UCP-mediated thermogenesis [1]. High-fat and sugar-enriched food are the core of NAFLD and metabolic syndrome risk factors, both of which directly modulate the intestinal and liver physiology, as well as the metabolic homeostasis of the whole body.

One of the main factors found to be affected by these metabolic disturbances is the intestinal microbiota (IM). The IM represents a physical entity composed of diverse bacterial, fungal and viral *phyla* which contribute to regulate the host immune system, help to extract nutrients from the diet and may even contribute to mood control [2]. IM is also related to the appearance and progression of several pathologies, among which is liver disease [3]. When not properly distributed, intestinal microbiota phyla composition may result in so-called dysbiosis. Dysbiosis represents an altered state of microbiota following antibiotic treatments, long periods of starvation or chronic ingestion of the Western diet (high-fat, high-fructose and processed meals), promoting the displacement of indigenous basal microbiota and inducing the proliferation of potential pathogenic harmful bacteria [4]. Pathogenic bacteria are thus able to traverse the gut mucus layer and eventually induce translocation into the bloodstream of pathogen-associated molecular patterns (PAMPs), including bacterial lipopolysaccharide (LPS) or other membrane-associated glycolipids [5].

As the portal circulation directly connects the intestinal tract and the liver, LPS glycolipids and intact bacteria may reach the liver tissue and consequently cause an inflammatory response, which along with increased lipolysis and carbohydrate and lipid metabolism disturbances, significantly contributes to the development and progression of liver steatosis (also known as fatty liver) and steatohepatitis (liver inflammation) [5].

NAFLD therapeutics comprises several pathways, including the modification of feeding habits, to increase physical activity and the ingestion of pre- or probiotics. Prebiotics are composed of complex plant-derived carbohydrates with soluble or insoluble dietary fiber [6]. It is known that certain intestinal indigenous bacteria are able to ferment the fiber in order to produce secondary metabolites with specific activities in eukaryotic cells. Currently, there is a long list of available prebiotics including the most common commercial fructans: fructo-oligosaccharides (FOS) and inulin [7]. Inulin-type fructans are present in several natural sources such as banana, garlic, onion, leek and other vegetables [7]. Inulin and FOS are resistant to digestion in the upper part of the intestine; instead, they enter the colon, where they serve as substrates to indigenous bacteria, which may convert this fiber into short-chain fatty acids (SCFAs) [7].

An improvement in liver disease after ingestion of the fructo-oligosaccharide inulin has been reported in several animal models, as well as in human subjects [8,9,10]. Among the mechanisms proposed to explain the beneficial effects of prebiotics on NAFLD and steatohepatitis is the direct effect on the de novo lipogenesis process [11], and down-regulation in the expression of lipogenesis-associated genes [12], as well as the increased synthesis of short-chain fatty acids, particularly acetate [13]. In addition, inulin may selectively promote the growth and activity of particular beneficial bacteria in the digestive tract, which in turn can promote indirect weight loss [14].

Here, we aim to address the effect of inulin treatment on the intestinal microbiota in a non-alcoholic fatty liver disease murine model. We observed that inulin treatment decreased lipid droplet deposition in the liver and significantly increased the presence of colonic *A. muciniphyla* and the *Prevotellaceae* family of bacteria, indicating that inulin is a viable method of tackling the liver-associated effects of metabolic syndrome.

## 2. Results

### 2.1. Body Weight Gain and Food Intake

**Figure 1:**
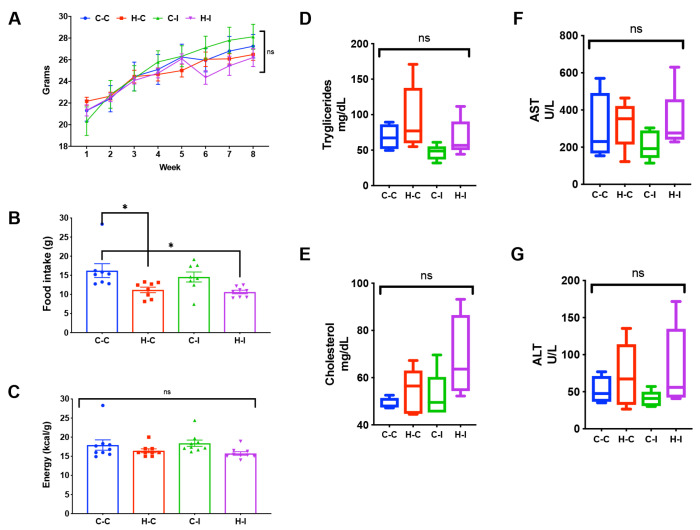
(**A**) Time-lapse of body weight gain in the different mice groups; (**B**,**C**) food and energy intake, respectively, at the end of the four weeks period of inulin treatment; Quantitation of serum triglycerides (**D**) and cholesterol (**E**) in mice of the indicated groups after four weeks of treatment. (**F**,**G**) Plots showing AST and ALT serum levels, in the four mice groups after the diet time period. Lines and bars in (**A**–**C**) indicate the mean ± SEM for each group, while boxplots in (**D**–**G**) indicate the median ± SD for each case. * *p* < 0.05; One-way ANOVA and Tukey test; ns, non-significant.

To gain insight on the direct or indirect effect of inulin supplementation on mice body weight, we calculated the difference between the initial and final body weight among the different groups. As can be seen in Figure 1A, body weight gain was similar between the control diet with cellulose (C-C) and the fat-enriched diet with cellulose (H-C) groups, independently of inulin supplementation. Moreover, we found that fat-enriched diet groups (H-C and H-I) had a significantly (*p* < 0.05) lower food intake compared to the control diet groups (Figure 1B); however, the whole energy intake (kcal/g) was not different among the groups (Figure 1C).

### 2.2. Biochemical Parameters

As metabolic syndrome has been associated with the consumption of a high-fat diet, eventually leading to an increase in triglycerides and cholesterol, along with the release of liver enzymes (AST, ALT, GGT), among other metabolic impairments in humans and rodent models, we measured some of these markers in serum samples of our mice. The analysis of triglyceride serum levels indicated that this parameter increased in the fat-enriched diet group (H-C) when compared with the control diet group (C-C), although the levels did not reach statistical significance (Figure 1D). Interestingly, inulin supplementation (H-I) successfully improved this increase, although not significantly (Figure 1D). This effect was also observed in the control diet group supplemented with inulin (C-I). Cholesterol levels on the other hand, also showed an increase in the high fat diet group (H-C), which was not avoided by inulin treatment (H-I) (Figure 1E). Control diets with (C-I) or without inulin (C-C) showed no differences in this parameter.

Liver enzymes showed that the fat-diet control group (H-C) had a strong increase in the ALT and AST circulating enzymes after the 8 weeks of feeding, although these were not significantly different against the control group (C-C). More importantly, these increases were diminished, although not to the levels of the control group, in the inulin-treated group (H-I) (Figure 1F,G). The control-diet groups, either with (C-I) or without inulin (C-C), showed no differences in both of these enzymes.

### 2.3. Inulin Supplementation Improves Liver Steatosis under High Fat Diet Regime

**Figure 2:**
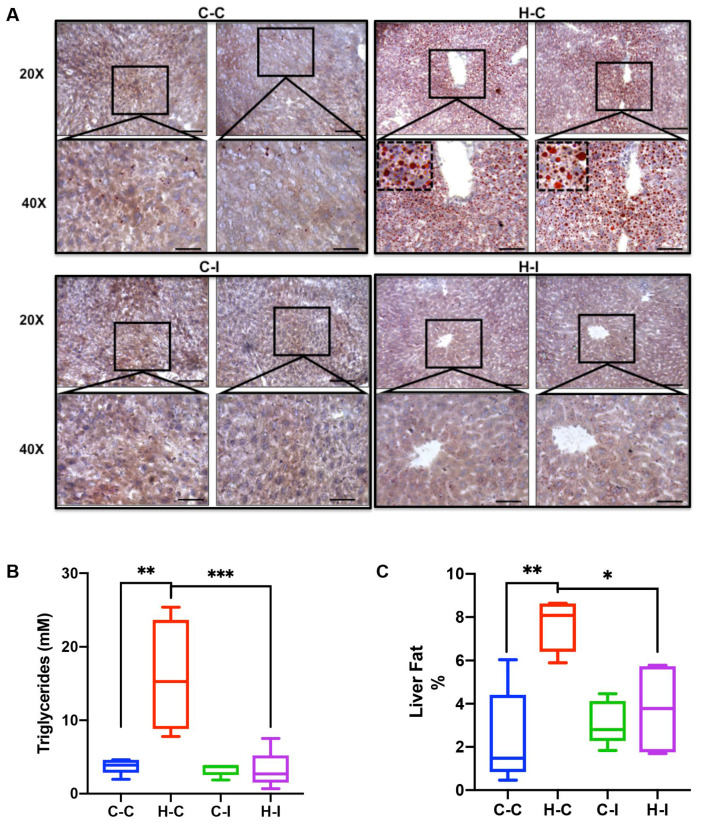
Inulin improves triglyceride accumulation on liver tissue after four weeks of treatment. (**A**) Liver histological sections were captured after Oil Red O staining as mentioned in materials and methods. Representative images from each group are showed for control groups without (C-C) or with inulin treatment (C-I), and fat-enriched groups (H-C and H-I). Micrographs show images of two mice-liver sections at 20× and magnifications of the squared regions at 40× below. The doted squares within 40× H-C group shows a 100× amplification of the corresponding image. (**B**) Boxplot showing the triglycerides concentration in liver tissue homogenates and (**C**) liver fat content in terms of percentage, for each diet group. * *p* < 0.05, ** *p* < 0.01 and *** *p* < 0.001, one-way ANOVA, Tukey’s test. Scale bar in panel A = 100 μm.

Fatty liver is a hallmark of metabolic syndrome and mice subject to the high-fat diet might develop this pathological feature over the course of the diet regime. Therefore, we sought to evaluate the effect of our different diets and inulin supplementation on mouse liver histology. We dissected liver tissue from all of the mice groups and performed Oil Red-O staining on liver histological sections. Here, we found that control-diet-fed animals were not histologically different, regardless of having received (C-I) or not received (C-C) the inulin treatment (Figure 2A). In contrast, the fat-enriched diet group (H-C) clearly showed a fatty liver phenotype, which was almost completely avoided when the animals were supplemented with inulin (H-I) (Figure 2A). Notably, inulin significantly improved the triglyceride content in the H-I group when compared with the H-C group (2.7 vs. 15.3 mM, respectively; *p* < 0.001) (Figure 2B). The fat liver content was also quantitated and, consistent with triglyceride data, showed that fat-enriched diet (H-C) increased the percentage of fat from 1.5% to 8% (*p* < 0.01), compared with control diet (C-C); this increase was avoided when inulin was introduced to the diet (8% in H-C to 3.8% in H-I; *p* < 0.05), although not to the levels observed in the control diet (C-C) group (Figure 2C).

### 2.4. Inulin Supplementation Re-Shapes the Gut Microbiota in High-Fat Diet Treated Mice

**Figure 3:**
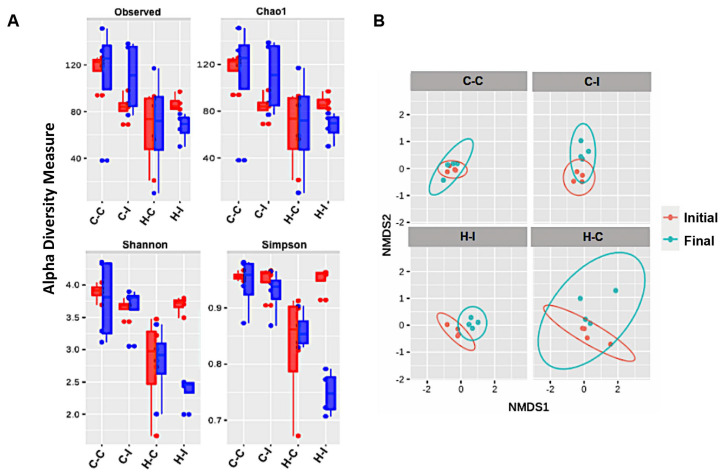
Inulin treatment modifies gut bacterial communities in mice subject to inulin supplementation. (**A**) Alpha diversity measures of cellulose control (C-C and H-C) or inulin supplemented (C-I and H-I) mice groups. The species enrichment is showed at the beginning (red boxes) and at the end of the supplement treatment (blue boxes). (**B**) Gut microbial non-metric multidimensional scale similarity plots indicating the differences among the four mice groups at the initial and final period of the experimental interventions.

As inulin is a prebiotic consisting of fermentable fiber and some of the beneficial effects of this fiber might be gained through the generation of secondary metabolites derived from the intestinal microbiota metabolism, we investigated the differences in the gut microbiota between cellulose- or inulin-supplemented diets. For this purpose, we performed sequencing of the bacterial 16S rRNA, from fecal-derived genomic DNA. Samples were rarefied to even sampling depths (Figure S1) before computing within-sample compositional α-diversity and between-sample β-diversity. In the first case, the data showed that richness and amplicon sequence variants (ASVs) abundance were not significantly different among the four mice groups (Figure 3A, upper panel); however, non-phylogenetic diversity and evenness or similarity indexes were significantly decreased in the fat-enriched diet group supplemented with inulin (H-I) when compared with their initial counterpart (*p* < 0.05) (Figure 3A, bottom panel; Supplementary Figure S1). Of note is that the fat-enriched diet groups, with or without inulin (H-C and H-I), lost a major part of their gut richness and evenness when compared with the control diet at the end of the study (Figure 3A).

**Figure 4:**
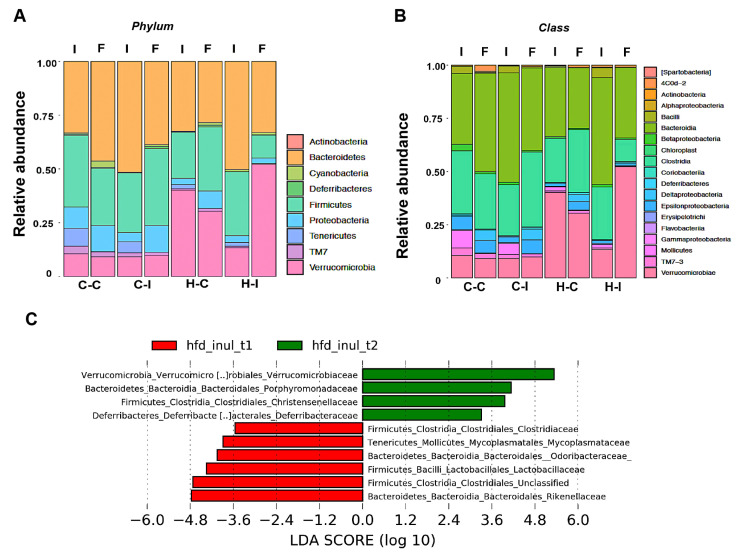
*Akkermansia* significantly increases in the gut microbiota after inulin supplementation. The corresponding relative abundance at the phylum (**A**) or class (**B**) level is presented for each group at the beginning (I) or at the end of eight weeks (F) of inulin supplementation (**C**) Linear discriminant analysis effect size is presented for the H-I group indicating the enterotype for this group before (red bars) or after (green bars) the eight weeks of treatment. Numbers indicate the correspondence fold change in log_10_ scale.

We then used β-diversity measures to determine if the bacterial communities were different among the groups at the end of the study. Here, we decided to use the non-phylogenetic Bray–Curtis distance in combination with the non-metric multidimensional scaling (NMDS) ordination, as a measure of similarity (dissimilarity) in our mice groups. We observed that bacterial communities are closely related at the beginning of the diet regime, as can be seen in Figure 3B. After inulin treatment, the gut microbial communities of the fat-enriched-diet groups were significantly shaped into independent enterotypes clearly separated from each other. These enterotypes were further analyzed by abundance and composition. We found that the most abundant members at the phyla level in all of the four groups were Bacteroidetes and Firmicutes; particularly, we observed that Verrucomicrobia was significantly augmented, at the expense of Firmicutes and *Protebacteria*, in the H-I group compared with the H-C diet at the end of the study (Figure 4, Phylum). In fact, Verrucomicrobia represented nearly 50% of the total enterotype in the H-I diet group at the end of the diet regime. *Deferribacteres* were also increased in this mice group. In contrast, the control diets showed that a small proportion of Tenericutes was lost at the end of inulin supplementation, while the Proteobacteria levels were predominant in both diets (C-C and C-I) (Figure 4, Phylum).

Further analysis indicated that *Deferribacteres* and *Verrucomicrobiae* were both increased at the class level in the H-I group at the end of inulin supplementation (Figure 4, Class). We performed a linear discriminant analysis (LDA) effect size (LEfSe) on this group and found, by microbial comparison at the family level, that *Verrucomicrobiaeceae*, *Porphyromonadaceae*, *Christenellaceae* and *Deferribacteraceae* displaced the *Clostridiaceae*, *Odoribacteriaceae*, *Lactobacillaceae* and *Rikenellaceae* phenotypes (Figure 4C).

**Figure 5:**
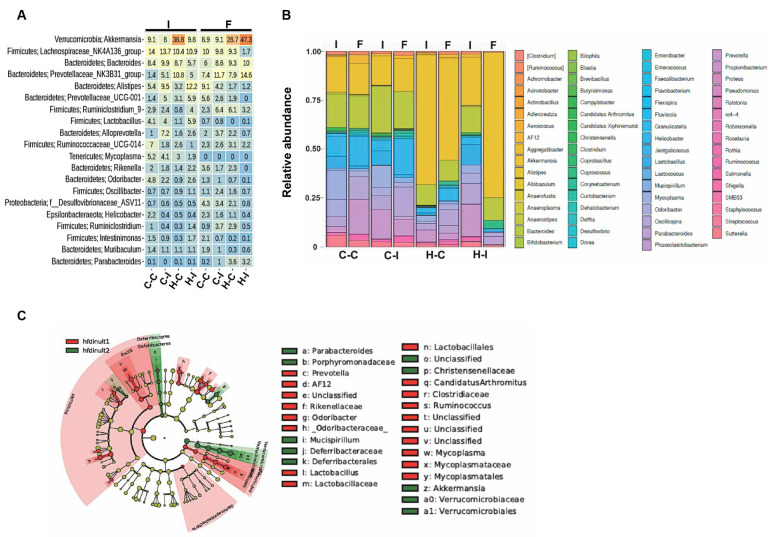
Representative features identified in the H-I mice group at the beginning (I) or end (F) of the inulin supplementation. (**A**) Fold change at the genus level in the four different mice groups. Numbers indicate percentage changes in each case respect its time counterpart and rows correspond to the genus associated. The genus level is plotted as the relative abundance in the four mice groups (**B**) or as the taxonomic differences focusing on the H-I group (**C**).

We then compared the fold percentage change at the genus level and found that *Akkermansia* significantly increased from near 10% to 47% in the H-I group at the end of the inulin supplementation (Figure 5A); a 30% net increase, compared with the 8% decrease in the control H-C diet group for this genus. We also found that the NK3B31 group increased by almost 10% and *Allistipes* decreased by 11% in the H-I group at the end of the experiment (Figure 5A,B). The overall enteroytpe of the H-I group at the genus level is shown in Figure 5C; it can be seen that Bacilli, *Gammaproteobacteria* and Tenericutes were replaced by *Deferribacteres*, *Parabacteroides* and *Verrucomicrobia* at the end of the inulin supplementation. According to the species-level analysis, A. muciniphila was the most predominant entity, along with *B. acidifaciens*, in the H-I group after supplementation (Supplementary Figure S2).

A correlation analysis showed that AST and ALT were positively associated with A. muciniphila in the H-I mice group at the end of inulin supplementation, but not with a significant difference (*p* = 0.37); meanwhile, cholesterol and albumin were negatively correlated, with a strong *p* value (*p* = 0.055) (Supplementary Figure S3A). A larger correlation analysis was also performed with other ASVs. Here, we found that *Rikenallaceae*, *S247* and *Odoribacter* were significantly and positively associated with the AST and ALT plasma levels, while Parabacteroides and *Prevotellaceae* are negatively correlated with those enzymes in this mice group, though not significantly (Supplementary Figure S3B). With respect to triglycerides and cholesterol, we observed that *Prevotellaceae* had a positive correlation (Pearson 0.81, *p* < 0.05) with cholesterol levels and *Parabecteroides* had a negative correlation (Pearson −0.95, *p* < 0.5) with triglycerides (Supplementary Figure S3B).

### 2.5. Effect of Inulin Supplementation on Gut and Liver Tight Junction Proteins

**Figure 6:**
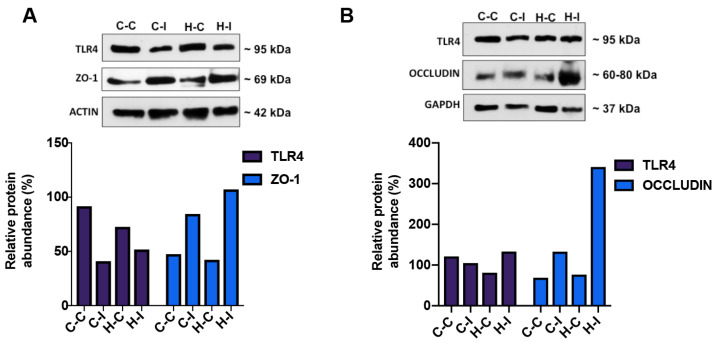
Inulin supplementation promotes the expression of tight junction proteins in the liver (**A**) or gut (**B**). Tissue representative blots from each mice group for toll-like receptor 4, ZO-1 and occludin proteins. Plots below indicate quantitation of the corresponding protein in fold percentage change.

As fat-enriched diet dysbiosis has been related to the disruption of gut physical barrier integrity, this might indirectly affect liver physiology; thus, we looked to explore if some of the gut or liver tight junction proteins were differentially expressed in response to the fat-enriched diet and the inulin treatment. Here, we observed that inulin supplementation in C-I and H-I mice groups was related to an increase in the expression of the zona occludens (ZO-1) protein in the liver at the end of the diet regimen, compared to those from the C-C or H-C groups; this increase was higher in the H-I group (Figure 6A). We found that Toll-like receptor 4 (TLR4) diminished in the H-I group when compared with the cellulose-treated group (H-C), suggesting a diminished pro-inflammatory response after inulin supplementation. The gut occludin expression showed an expression pattern similar to that of ZO-1, although was significantly increased to a greater extent after inulin supplementation in the H-I mice group, while TLR4 did not change significantly in any of the four mice groups (Figure 6B). Together, these data suggest that inulin supplementation might contribute to restore the epithelial physical barrier disturbances associated with high-fat diets. Whether this effect is related to the resident microbiota modification, and to reducing intestinal permeability through tight junction protein regulation, is a matter of debate.

### 2.6. Metagenome Inference

Our data indicate that *Verrucomicrobiaceae*, specifically *Akkermansia*, is the most abundant entity at the genus level in response to the inulin supplementation of the H-I mice; therefore, we sought to estimate the bacterial gene pathway abundances in this group by means of the PICRUSt protocol [15]. Our data indicate that some of the gene pathways enriched in the H-I group at the end of inulin treatment were the branched chain amino acids degradation, fatty acid and biotin metabolism, and the glutathione and secondary metabolites processing, when comparing the initial and final treatment (Supplementary Figure S4). However, one of the most interesting modified pathway abundances was that of propanoate metabolism, which has been related to inulin consumption.

## 3. Discussion

We show here that inulin supplementation in a murine model of hepatic steatosis, induced by fat consumption, decreases fatty liver deposition, and modifies the intestinal microbiota to a specific enterotype. We found that mice fed with a fat-enriched diet did not show a significant weight gain as we expected. However, mice did show the histological characteristics of a fatty liver, indicating that this strategy may be considered a “metabolically obese, normal weight” phenotype, a term coined after observations of metabolic dysfunction in the absence of obesity [16]. This phenotype seems to have high prevalence among humans [16]. Here, we phenocopied this metabolic state and found that metabolically obese mice supplemented with inulin showed an improvement in their hepatic steatosis, as well as a tendency towards decreased AST and ALT liver enzyme levels, and to a lesser extent diminished serum triglyceride and cholesterol levels. It should be noted that inulin supplementation did not seem to exert a direct effect on weight gain, as indicated in Figure 1. 

As inulin is considered a prebiotic dietary fiber supplement, with healthy properties such as lipid metabolism improvement, stool deposition frequency and a bifidogenic effect, capable of modifying the relative abundance of different bacteria within the gastrointestinal tract, it is not unexpected that its chronic consumption might have beneficial effects in our metabolically obese mice. Indeed, we observed that inulin supplementation completely modifies the mice gut microbiota from a predominant Firmicutes, Bacteroidetes and Tenericutes phyla enterotype to a Bacteroidetes and Verrucomicrobia phyla enterotype, especially in mice under a fat-enriched diet regime. A detailed view of the sequencing data showed that the *Verrucomicrobiaceae* family significantly increased by more than 5-fold compared with the baseline relative abundance in the H-I group, similar to previously reported data that suggested that inulin and fructo-oligosaccharides at different doses modulate mice intestinal microbiota, promoting an increase in *Verrucomicrobia* [17].

There are several reports indicating that *A. muciniphila* intestinal abundance is promoted by the ingestion of the prebiotic fiber inulin in mammals [18,19,20]. Seminal papers from M. Roberfroid, N. Delzenne and P. Cani at the University of Louvain in Belgium have shown the synergistic effects of prebiotics when administered along with probiotics. These teams of researchers have evidenced that this organism synthesizes and releases enzymes that help to regulate mucin, the main intestinal mucus layer precursor [21]. It is also known that *A. muciniphila* adheres to the enterocytes and helps to maintain the integrity of the gut inner epithelium [22]. The mucus layer and the microbiota associated with it cooperate to protect the physical integrity of the lining intestines from injury that might lead to the loss of the impermeability of this epithelium. Therefore, the loss of the physical barrier has been related to chronic diseases such as obesity, diabetes and inflammatory processes. It has also been suggested that *A. muciniphila* protects against the inflammatory response elicited by endotoxemia, a condition raised by the loss of the physical barrier [23]. Decreasing levels or the loss of *A. muciniphila* are related to an increased risk of obesity, metabolic syndrome and type 2 diabetes [24]. We observed that our mice started with a low relative abundance of *Verrucomicrobia*, which significantly increased at the end of inulin supplementation, especially in the fat-enriched-diet group, suggesting that inulin is able to increase the abundance of this *phylum*. Despite the major role that *A. muciniphila* might have in our findings, it is also plausible that other bacteria may contribute to the beneficial effects of inulin supplementation. There is clinical evidence suggesting that oligofructose and inulin increase the relative abundance of *Bifidobacteria* in the stool of healthy human volunteers [25,26]. Our 16S data do not show an enrichment of *Bifidobacteria*, but instead of the *Deferribacteraceae* and *Christensenelaceae* families; the latter has been proposed as a fermentative due to its abundance in the distal colon [27]. 

As mentioned before, the inflammatory response as a consequence of the loss of gut epithelial barrier function is a common finding in metabolic syndrome, and there are several proteins that could be used to evaluate both. Toll-like receptor 4 (TLR4) is one such protein related to the inflammatory pathway [5], while zone occludens 1 (ZO-1) and occludin are related to the gut epithelium physical barrier integrity [5]. In the first case, it has been suggested that TLR4 is activated by exogenous or endogenous signals, such as lipopolysaccharides (LPS), considered the main pathogen-associated molecular patterns (PAMPs) or damage-associated molecular pattern molecules (DAMPs). Both LPS and DAMPs strongly activate the Toll-like receptors (TLRs) present in the gut or liver, especially in the Kupffer cells (which play a role in metabolic endotoxemia). The pro-inflammatory IKKκ-NFκB-mediated signaling pathway thus induces the gene expression, synthesis and release of pro-inflammatory cytokines [28]. Here, we determined the level of TLR4 protein expression in both liver and gut of our four mice groups. We observed that liver TLR4 expression levels in control groups (C-C and H-C) were similar, but after inulin supplementation these levels diminished (see Figure 6A), suggesting that inulin is either directly regulating the expression of this receptor or modifying the gut microbiota, which may indirectly modify its expression. Surprisingly, the intestinal TLR4 expression levels were not significantly affected by the fat-enriched diet or inulin supplementation. It is probable that the fatty acids in the diet did not reach a threshold required within the gut to activate the TLR4 gene expression, or that the inflammatory response was not strong enough to alter its regulation at the protein level. Another receptor may have been activated, such as TLR2 [29,30], a protein we did not measure.

The tight junction proteins, ZO-1 and occludin, are important mediators of cell-cell communication by means of ion, solute and peptide movements. These two membrane-bound proteins are subject to basal regulation by post-translational modifications such as PKC-mediated phosphorylation [5], but proinflammatory cytokines such as tumor-necrosis factor alpha (TNF-α) or interferon gamma (IFN-γ) can also alter the physical properties of ZO-1 and occludin and increase their permeability [31,32]. As mentioned before, it is known that the gut microbiota is the main source of lipopolysaccharide (LPS), a bacterial cell wall component able to activate TLR4, which in turn leads to a proinflammatory state known to alter the ZO-1 and occludin protein assembly in the intestinal epithelium, generating metabolic endotoxemia (LPS translocation to the luminal side) [5,33]. It has been reported that the down-regulation of ZO-1 and occludin proteins is correlated with disease [34,35], and here, we found a marked increase in the liver and intestinal whole extracts, respectively, of the inulin-supplemented groups (C-I or H-I) when compared with their controls (C-C or H-C), suggesting that inulin counteracted the impairment of the mucosal permeability induced by the fat-enriched diet and could also contribute to maintain the physical barrier function and, consequently, the metabolic endotoxemia and inflammatory state.

Finally, we found that metagenome inference indicates that the propionate metabolism was one of the most intriguing gene pathways enriched in our mice with inulin supplementation under a fat-enriched diet. Inulin might contribute to the citric acid cycle through propionate, which is considered a gluconeogenic short-chain fatty acid [36]; however, it is well known that *A. muciniphila* can degrade mucin, a complex glycoprotein secreted by the gut epithelial cells in mammals [37,38] and generate short-chain fatty acids such as propionate and acetate, which in turn stimulate the epithelial cells to further synthesize and excrete more mucin, or to stimulate the growth of other commensal beneficial butyrate-producing bacteria within the intestine in a complex “cross-feeding” mechanism [39,40] In this context, it has been reported that oral administrations of fructo-oligosaccharides (FOS) such as inulin (a polysaccharide fructan) are able to act as a prebiotic, increasing the *A. muciniphila* abundance in diet-induced or genetically induced obese mice [41,42]. Human studies indicates that the so-called FODMAP (fermentable oligo-, di- and monosaccharides and polyols) diet may increase the levels of *A. muciniphila* in healthy subjects or in subjects with Crohn´s disease [43,44]. A more recent study in which a group of overweight and obese volunteers were supplemented orally with live or pasteurized *A. muciniphila* (10^7^ bacteria) showed that, after three months of treatment, volunteers improved their insulin resistance and insulinemia, and had reduced levels of plasma cholesterol, as well as LDH, AST and GGT hepatic enzymes [45]; the authors suggest that *A. muciniphila* may indirectly contribute to reinforce gut barrier function, thus impeding LPS to translocate to the portal blood circulation. The production of propionate or acetate by *A. muciniphila* seems the most likely mechanism to be mediating these effects, although additional studies are needed to complement these data. 

Additional evidence shows that propionate alters hepatic metabolism processes to reduce lipid content [46] however, hepatic metabolic processing of propionate seems to be conditioned according to the status of energy balance [11]. Previous data indicate that dysbiosis has an impact in the development of certain diseases such hepatic steatosis. In this context, the intestine-liver axis is a reference to the functional and anatomical association between both organs, and it implies the route of molecules associated with the gut microbiota from the intestine to the liver [47].

## 4. Materials and Methods

### 4.1. Animals and Treatments

The animal care and research committee read and approved all the animal procedures of this protocol (CICUAL: GAS-1850-16/17-1). A total of forty C57/BL-6N eight-weeks old male mice were randomly split into four different groups of ten animals each. As inulin is a fermentable fructo-oligosaccharide, we decided to use the non-fermentable fiber cellulose as control. Mice groups (ten mice per group/five per cage) were divided as follows: (1)it is not chow-diet (D12450K, 10% kcal from fat, Research Diets, New Brunswick, NJ, USA) with cellulose (10% *w*/*w*, Sigma Aldrich, EdoMex, México) (C-C), or (2) inulin (10% *w*/*w*, Azelis Megafarma, CDMX, México) (C-I); (3) fat-enriched diet (D12451, 45% kcal from fat, Research Diets), supplemented with cellulose (H-C) or (4) fat-enriched diet supplemented with inulin (H-I). The total amount of fiber in each diet, considering the reported manufacturer diet-formula content and the added inulin or cellulose was 15% to 16%. All of these groups were maintained for eight weeks in the animal core facility at controlled temperature and humidity in a 12-h/12-h dark/light cycles. Diets and water were ad libitum. Food intake and body weight gain were registered every other day. After eight weeks, mice were anesthetized, and peripheral blood samples collected by cardiac puncture in EDTA-treated tubes. Plasma was recovered after centrifugation and stored at −20 °C until use. In addition, the liver was dissected for histology or whole protein extraction (see below).

### 4.2. Biochemical Parameters

Plasma samples were used to determine liver enzymes alanine aminotransferase (ALT), aspartate aminotransferase (AST), as well as cholesterol (CHOL), albumin (ALB), and triglycerides (TG) using specific reagents for Cobas c111 device (Roche Diagnostics GmbH, Mannheim, Germany), following the manufacturer instructions.

### 4.3. Histology

Liver tissue was processed for histology as previously described [48]. Briefly, for Oil-Red-O staining the excised liver was used to obtain fragments that were frozen in OCT; then, 8µm sections were obtained with a pedestal cryostat that where briefly rinsed in distilled water, followed by few seconds in isopropyl alcohol and then submerged in Oil-Red-O stain by 20 min. After that time, tissue sections were rinsed and counterstained with Haematoxylin and mounted in glycerine jelly. Stained slides were imaged under a Leica DM750 microscope (Leica, Wetzlar, Germany) at 20 and 40× magnification. Lipid droplet quantification was performed on ImageJ software (V1.53c, NIH, Bethesda, MD, USA) [49].

### 4.4. Quantitation of Liver Fat and Triglycerides

Liver fat was quantitated by the improved Folch method recently reported with minor modifications [50]. Briefly, 150 mg of liver tissue section was mechanically homogenized in chloroform:methanol (2:1) solution. Samples were then agitated overnight at room temperature. Then, samples were centrifuged at 3000× *g* for 10 min in order to collect the supernatant. Afterwards, 400 μL of 0.9% NaCl was added to the recovered supernatant and the mixture was vortexed and centrifuged at 2500× *g* 10 min. The upper phase was discarded and the residual interface was rinsed twice with 50% methanol:H_2_O (*v*/*v*). The remaining fatty phase was collected and evaporated in the speed-vac apparatus at 45 °C during 2 h. The fat weight was recorded, and the hepatic fat content was calculated as percentage of the wet liver weight. 

Finally, liver triglycerides (TG) were determined according to the guidelines of a commercial colorimetric kit (Triglyceride Quantification Kit K622-100, Bio Vision, CA, USA) using 100 mg of liver tissue.

### 4.5. Faecal Genomic DNA Extraction

Fecal samples from all of the mice groups were collected directly from mice rectum, at the beginning and at the end of the eight weeks of the fiber supplementation. Here, because of technical reasons, we decided to pool two-mice fecal samples in order to get five samples in each group. Samples were collected in sterilized containers and stored at −70 °C until use. Genomic DNA (gDNA) was extracted following the Qiamp DNA Stool Mini Kit guidelines (Qiagen, Germantown, MD, USA) and quantified in a Nanodrop apparatus (Invitrogen, Carlsbad, CA, USA). The relationship 260/280 nm above 1.5 was used as a measure of purity. The gDNA samples were stored at −20°C until library preparation.

### 4.6. 16S rRNA Gene Sequencing and Data Analysis

The gDNA was processed following the 16S library preparation workflow (2 × 250 bp reads lengths; Illumina, San Diego, CA, USA). Briefly: gDNA (10 ng) was PCR-amplified using the V3-V4 16S rRNA hyper-variable region primer-pairs 341F/805R, as previously described [51]. Amplicons were quantified using the dsDNA-HS kit (Invitrogen) on a Qubit device (Life Technologies, Carlsbad, CA, USA). The products were used to generate the sequencing library using the Illumina barcodes. Library was normalized and pooled to load into the NextSeq flow cell (Nextera XT, 250 × 2 reads, Illumina). Raw data (fastq files) were processed according to the DADA2 pipeline (v1.16) [52]. Briefly: raw paired-end fastq files were inspected and filter with a maximum error rate of 2 with no N´s allowed and a truncation quality read threshold of 2. Afterwards, denoising was applied to infer amplicon sequence variants (ASV) before merging paired reads to construct the sequence table. The ASV table was further filtered for chimeras using the consensus method. Taxonomy was determined using the chimera filtered ASV table and the naïve Bayesian classifier method using the Silva v132 trained data set [53]. Species assignment was performed with the Silva species assignment script. Once the ASV and taxonomy tables were generated, both were exported and analyzed using Phyloseq [54]. Phyla that were under-represented (<1 read per sample) or with least than 5% of prevalence threshold in all samples, as well as those reads corresponding to chloroplasts, mitochondria or eukaryotes, were discarded. Alpha and beta diversity were estimated with Observed, Shannon and Simpson indexes or the phylogeny-independent multivariate analysis based on Bray–Curtis distance and the non-metric multidimensional scaling ordination method (NMDS), respectively. Microbial composition and abundance plotting was performed with fantaxtic package (V 0.1.0) for phyloseq and ggplot2 (Tydiverse) in R. A linear discriminant analysis effect size was performed using LEfSe module [55], in the Galaxy platform [56]. Metagenomic estimates were inferred using PICRUSt [57], and metabolic data plots were generated using the LEfSe module in Galaxy platform.

### 4.7. Western Blotting

Liver tissue extracts were prepared as described [58], and blots were probed with anti-TLR4 (1:1000) (SC-293072, Santa-Cruz Biotechnology, Dallas, TX, USA), anti-ZO-1 (1:1000) (SC-33725, Santa-Cruz Biotechnology), anti-Occludin (1:500) (SC-133256, Santa-Cruz Biotechnology), and anti-Actin (1:5000) (SC-1616, Santa-Cruz Biotechnology,) or anti-Gapdh (1:3000) (SC-47724, Santa-Cruz Biotechnology), as loading controls. Blots were developed with chemiluminescence reagent (Immobilon HRP-substrate reagent; Millipore, Burlington, MA, USA) and the corresponding images were acquired with the ChemiDoc-MP Imaging detection system (BioRad; CDMX, México). Images were further processed for densitometry analysis using ImageJ software (V 1.53c) [49]. 

### 4.8. Statistical Analysis

Data are presented as means ± SE. median (interquartile range), or percentage. Comparison among groups was performed with two-way ANOVA, using the non-parametric post-hoc Tukey’s, or Bonferroni, as needed. *p*-values ≤ 0.05 were considered statistically significant. All statistical analyses were performed using Prism software (GraphPad Software v8; San Diego, CA, USA).

## 5. Conclusions

In this study we found that a group of mice subject to a fat-enriched diet developed a low-grade liver steatosis. These mice also showed significant changes in their gut microbiota. However, inulin supplementation over 8 weeks was able to significantly reduce the liver lipid droplet deposition and modify the gut bacterial composition, favoring in particular the abundance of *Parabacteroides, Deferribacteeres* and *Verrucomicrobia*, compared with those receiving a non-fermentable fiber such as cellulose. Altogether, our data indicate that inulin supplementation is able to improve the fatty liver-related diet-induced damage.

## Data Availability

Data are available upon reasonable request.

## Supplementary Materials

The following supporting information can be downloaded at: https://www.mdpi.com/article/10.3390/ijms23020991/s1.

## References

[1] Gadde K.M., Martin C.K., Berthoud H.-R., Heymsfield S.B. (2018). Obesity: Pathophysiology and Management. J. Am. Coll. Cardiol..

[2] Adak A., Khan M.R. (2019). An Insight into Gut Microbiota and Its Functionalities. Cell. Mol. Life Sci..

[3] Koopman N., Molinaro A., Nieuwdorp M., Holleboom A.G. (2019). Review Article: Can Bugs Be Drugs? The Potential of Probiotics and Prebiotics as Treatment for Non-Alcoholic Fatty Liver Disease. Aliment. Pharmacol. Ther..

[4] Carding S., Verbeke K., Vipond D.T., Corfe B.M., Owen L.J. (2015). Dysbiosis of the Gut Microbiota in Disease. Microb. Ecol. Health Dis..

[5] Mohammad S., Thiemermann C. (2021). Role of Metabolic Endotoxemia in Systemic Inflammation and Potential Interventions. Front. Immunol..

[6] Holscher H.D. (2017). Dietary Fiber and Prebiotics and the Gastrointestinal Microbiota. Gut Microbes.

[7] Flamm G., Glinsmann W., Kritchevsky D., Prosky L., Roberfroid M. (2001). Inulin and Oligofructose as Dietary Fiber: A Review of the Evidence. Crit. Rev. Food Sci. Nutr..

[8] Daubioul C.A., Horsmans Y., Lambert P., Danse E., Delzenne N.M. (2005). Effects of Oligofructose on Glucose and Lipid Metabolism in Patients with Nonalcoholic Steatohepatitis: Results of a Pilot Study. Eur. J. Clin. Nutr..

[9] Zhang J., Li Y., Xiao G., Li Y., Xie H., Chen Y. (2021). Fructooligosaccharides Enhance the Therapeutic Effect of Xiao-Zhi-Fang on Non-Alcoholic Fatty Liver Disease via Regulating Intestinal Flora. Liver Res..

[10] Takai A., Kikuchi K., Ichimura M., Tsuneyama K., Moritoki Y., Matsumoto K., Tsunashima H., Onda T., Kuniyoshi N., Nariyama T. (2020). Fructo-Oligosaccharides Ameliorate Steatohepatitis, Visceral Adiposity, and Associated Chronic Inflammation via Increased Production of Short-Chain Fatty Acids in a Mouse Model of Non-Alcoholic Steatohepatitis. BMC Gastroenterol..

[11] Chambers E.S., Byrne C.S., Rugyendo A., Morrison D.J., Preston T., Tedford C., Bell J.D., Thomas L., Akbar A.N., Riddell N.E. (2019). The Effects of Dietary Supplementation with Inulin and Inulin-Propionate Ester on Hepatic Steatosis in Adults with Non-Alcoholic Fatty Liver Disease. Diabetes Obes. Metab..

[12] Sugatani J., Osabe M., Wada T., Yamakawa K., Yamazaki Y., Takahashi T., Ikari A., Miwa M. (2008). Comparison of Enzymatically Synthesized Inulin, Resistant Maltodextrin and Clofibrate Effects on Biomarkers of Metabolic Disease in Rats Fed a High-Fat and High-Sucrose (Cafeteria) Diet. Eur. J. Nutr..

[13] Chen H.-T., Huang H.-L., Li Y.-Q., Xu H.-M., Zhou Y.-J. (2020). Therapeutic Advances in Non-Alcoholic Fatty Liver Disease: A Microbiota-Centered View. World J. Gastroenterol..

[14] Parnell J.A., Reimer R.A. (2012). Prebiotic Fibres Dose-Dependently Increase Satiety Hormones and Alter Bacteroidetes and Firmicutes in Lean and Obese JCR:LA-Cp Rats. Br. J. Nutr..

[15] Chassard C., Delmas E., Robert C., Bernalier-Donadille A. (2010). The Cellulose-Degrading Microbial Community of the Human Gut Varies According to the Presence or Absence of Methanogens. FEMS Microbiol. Ecol..

[16] Ding C., Chan Z., Magkos F. (2016). Lean, but Not Healthy: The ‘Metabolically Obese, Normal-Weight’ Phenotype. Curr. Opin. Clin. Nutr. Metab. Care.

[17] Zhu L., Qin S., Zhai S., Gao Y., Li L. (2017). Inulin with Different Degrees of Polymerization Modulates Composition of Intestinal Microbiota in Mice. FEMS Microbiol. Lett..

[18] Biruete A., Cross T.-W.L., Allen J.M., Kistler B.M., de Loor H., Evenepoel P., Fahey G.C., Bauer L., Swanson K.S., Wilund K.R. (2021). Effect of Dietary Inulin Supplementation on the Gut Microbiota Composition and Derived Metabolites of Individuals Undergoing Hemodialysis: A Pilot Study. J. Ren. Nutr..

[19] Bao T., He F., Zhang X., Zhu L., Wang Z., Lu H., Wang T., Li Y., Yang S., Wang H. (2020). Inulin Exerts Beneficial Effects on Non-Alcoholic Fatty Liver Disease via Modulating Gut Microbiome and Suppressing the Lipopolysaccharide-Toll-Like Receptor 4-Mψ-Nuclear Factor-ΚB-Nod-Like Receptor Protein 3 Pathway via Gut-Liver Axis in Mice. Front. Pharmacol..

[20] Xia B., Wu W., Zhang L., Wen X., Xie J., Zhang H. (2021). Gut Microbiota Mediates the Effects of Inulin on Enhancing Sulfomucin Production and Mucosal Barrier Function in a Pig Model. Food Funct..

[21] Corazziari E.S. (2009). Intestinal Mucus Barrier in Normal and Inflamed Colon. J. Pediat. Gastroenterol. Nutr..

[22] Reunanen J., Kainulainen V., Huuskonen L., Ottman N., Belzer C., Huhtinen H., de Vos W.M., Satokari R. (2015). Akkermansia Muciniphila Adheres to Enterocytes and Strengthens the Integrity of the Epithelial Cell Layer. Appl. Environ. Microbiol..

[23] Li J., Lin S., Vanhoutte P.M., Woo C.W., Xu A. (2016). Akkermansia Muciniphila Protects Against Atherosclerosis by Preventing Metabolic Endotoxemia-Induced Inflammation in Apoe−/− Mice. Circulation.

[24] Yassour M., Lim M.Y., Yun H.S., Tickle T.L., Sung J., Song Y.-M., Lee K., Franzosa E.A., Morgan X.C., Gevers D. (2016). Sub-Clinical Detection of Gut Microbial Biomarkers of Obesity and Type 2 Diabetes. Genome Med..

[25] Gibson G.R., Beatty E.R., Wang X., Cummings J.H. (1995). Selective Stimulation of Bifidobacteria in the Human Colon by Oligofructose and Inulin. Gastroenterology.

[26] Bouhnik Y., Vahedi K., Achour L., Attar A., Salfati J., Pochart P., Marteau P., Flourié B., Bornet F., Rambaud J.-C. (1999). Short-Chain Fructo-Oligosaccharide Administration Dose-Dependently Increases Fecal Bifidobacteria in Healthy Humans. J. Nutr..

[27] Waters J.L., Ley R.E. (2019). The Human Gut Bacteria Christensenellaceae Are Widespread, Heritable, and Associated with Health. BMC Biol..

[28] Molteni M., Gemma S., Rossetti C. (2016). The Role of Toll-Like Receptor 4 in Infectious and Noninfectious Inflammation. Mediat. Inflamm..

[29] Shi H., Kokoeva M.V., Inouye K., Tzameli I., Yin H., Flier J.S. (2006). TLR4 Links Innate Immunity and Fatty Acid–Induced Insulin Resistance. J. Clin. Investig..

[30] Cani P.D., de Vos W.M. (2017). Next-Generation Beneficial Microbes: The Case of Akkermansia Muciniphila. Front. Microbiol..

[31] Ma T.Y., Iwamoto G.K., Hoa N.T., Akotia V., Pedram A., Boivin M.A., Said H.M. (2004). TNF-α-Induced Increase in Intestinal Epithelial Tight Junction Permeability Requires NF-ΚB Activation. Am. J. Physiol. Gastrointest. Liver Physiol..

[32] Smyth D., Phan V., Wang A., McKay D.M. (2011). Interferon-γ-Induced Increases in Intestinal Epithelial Macromolecular Permeability Requires the Src Kinase Fyn. Lab. Invest..

[33] Lee B., Moon K.M., Kim C.Y. (2018). Tight Junction in the Intestinal Epithelium: Its Association with Diseases and Regulation by Phytochemicals. J. Immunol. Res..

[34] Hwang I., An B.S., Yang H., Kang H.S., Jung E.M., Jeung E.B. (2013). Tissue-Specific Expression of Occludin, Zona Occludens-1, and Junction Adhesion Molecule A in the Duodenum, Ileum, Colon, Kidney, Liver, Lung, Brain, and Skeletal Muscle of C57BL Mice. J. Physiol. Pharmacol..

[35] Xin D., Zong-Shun L., Bang-Mao W., Lu Z. (2014). Expression of Intestinal Tight Junction Proteins in Patients with Non-Alcoholic Fatty Liver Disease. Hepatogastroenterology.

[36] Verbrugghe A., Hesta M., Gommeren K., Daminet S., Wuyts B., Buyse J., Janssens G.P. (2009). Oligofructose and Inulin Modulate Glucose and Amino Acid Metabolism through Propionate Production in Normal-Weight and Obese Cats. Br. J. Nutr..

[37] Collado M.C., Derrien M., Isolauri E., de Vos W.M., Salminen S. (2007). Intestinal Integrity and Akkermansia Muciniphila, a Mucin-Degrading Member of the Intestinal Microbiota Present in Infants, Adults, and the Elderly. Appl. Environ. Microbiol..

[38] Derrien M., Collado M.C., Ben-Amor K., Salminen S., de Vos W.M. (2008). The Mucin Degrader Akkermansia Muciniphila Is an Abundant Resident of the Human Intestinal Tract. Appl. Environ. Microbiol..

[39] Hansson G.C. (2020). Mucins and the Microbiome. Annu. Rev. Biochem..

[40] Hagi T., Belzer C. (2021). The Interaction of Akkermansia Muciniphila with Host-Derived Substances, Bacteria and Diets. Appl. Microbiol. Biotechnol..

[41] Everard A., Belzer C., Geurts L., Ouwerkerk J.P., Druart C., Bindels L.B., Guiot Y., Derrien M., Muccioli G.G., Delzenne N.M. (2013). Cross-Talk between Akkermansia Muciniphila and Intestinal Epithelium Controls Diet-Induced Obesity. Proc. Natl. Acad. Sci. USA.

[42] Everard A., Lazarevic V., Derrien M., Girard M., Muccioli G.G., Neyrinck A.M., Possemiers S., Holle A.V., François P., de Vos W.M. (2011). Responses of Gut Microbiota and Glucose and Lipid Metabolism to Prebiotics in Genetic Obese and Diet-Induced Leptin-Resistant Mice. Diabetes.

[43] Halmos E.P., Christophersen C.T., Bird A.R., Shepherd S.J., Gibson P.R., Muir J.G. (2015). Diets That Differ in Their FODMAP Content Alter the Colonic Luminal Microenvironment. Gut.

[44] Halmos E.P., Christophersen C.T., Bird A.R., Shepherd S.J., Muir J.G., Gibson P.R. (2016). Consistent Prebiotic Effect on Gut Microbiota with Altered FODMAP Intake in Patients with Crohn’s Disease: A Randomised, Controlled Cross-Over Trial of Well-Defined Diets. Clin. Transl. Gastroenterol..

[45] Depommier C., Everard A., Druart C., Plovier H., Van Hul M., Vieira-Silva S., Falony G., Raes J., Maiter D., Delzenne N.M. (2019). Supplementation with Akkermansia Muciniphila in Overweight and Obese Human Volunteers: A Proof-of-Concept Exploratory Study. Nat. Med..

[46] Lin Y., Vonk R.J., Slooff M.J.H., Kuipers F., Smit M.J. (1995). Differences in Propionate-Induced Inhibition of Cholesterol and Triacylglycerol Synthesis between Human and Rat Hepatocytes in Primary Culture. Br. J. Nutr..

[47] Porras D., Nistal E., Martínez-Flórez S., Olcoz J.L., Jover R., Jorquera F., González-Gallego J., García-Mediavilla M.V., Sánchez-Campos S. (2019). Functional Interactions between Gut Microbiota Transplantation, Quercetin, and High-Fat Diet Determine Non-Alcoholic Fatty Liver Disease Development in Germ-Free Mice. Mol. Nutr. Food Res..

[48] Rodriguez-Rodriguez C., Torres N., Gutierrez-Uribe J.A., Noriega L.G., Torre-Villalvazo I., Leal-Diaz A.M., Antunes-Ricardo M., Marquez-Mota C., Ordaz G., Chavez-Santoscoy R.A. (2015). The Effect of Isorhamnetin Glycosides Extracted from Opuntia Ficus-Indica in a Mouse Model of Diet Induced Obesity. Food Funct..

[49] Schindelin J., Arganda-Carreras I., Frise E., Kaynig V., Longair M., Pietzsch T., Preibisch S., Rueden C., Saalfeld S., Schmid B. (2012). Fiji: An Open-Source Platform for Biological-Image Analysis. Nat. Methods.

[50] Mopuri R., Kalyesubula M., Rosov A., Edery N., Moallem U., Dvir H. (2021). Improved Folch Method for Liver-Fat Quantification. Front. Vet. Sci..

[51] Klindworth A., Pruesse E., Schweer T., Peplies J., Quast C., Horn M., Glockner F.O. (2013). Evaluation of General 16S Ribosomal RNA Gene PCR Primers for Classical and Next-Generation Sequencing-Based Diversity Studies. Nucleic Acids Res..

[52] Callahan B.J., McMurdie P.J., Rosen M.J., Han A.W., Johnson A.J.A., Holmes S.P. (2016). DADA2: High-Resolution Sample Inference from Illumina Amplicon Data. Nat. Methods.

[53] Quast C., Pruesse E., Yilmaz P., Gerken J., Schweer T., Yarza P., Peplies J., Glöckner F.O. (2013). The SILVA Ribosomal RNA Gene Database Project: Improved Data Processing and Web-Based Tools. Nucleic Acids Res..

[54] McMurdie P.J., Holmes S. (2013). Phyloseq: An R Package for Reproducible Interactive Analysis and Graphics of Microbiome Census Data. PLoS ONE.

[55] Segata N., Izard J., Waldron L., Gevers D., Miropolsky L., Garrett W.S., Huttenhower C. (2011). Metagenomic Biomarker Discovery and Explanation. Genome Biol..

[56] Afgan E., Baker D., Batut B., van den Beek M., Bouvier D., Cech M., Chilton J., Clements D., Coraor N., Gruning B.A. (2018). The Galaxy Platform for Accessible, Reproducible and Collaborative Biomedical Analyses: 2018 Update. Nucleic Acids Res..

[57] Langille M.G., Zaneveld J., Caporaso J.G., McDonald D., Knights D., Reyes J.A., Clemente J.C., Burkepile D.E., Vega Thurber R.L., Knight R. (2013). Predictive Functional Profiling of Microbial Communities Using 16S RRNA Marker Gene Sequences. Nat. Biotechnol..

[58] Perez-Monter C., Martinez-Armenta M., Miquelajauregui A., Furlan-Magaril M., Varela-Echavarria A., Recillas-Targa F., May V., Charli J.L., Perez-Martinez L. (2011). The Kruppel-like Factor 4 Controls Biosynthesis of Thyrotropin-Releasing Hormone during Hypothalamus Development. Mol. Cell Endocrinol..

