# Supplementary material for: Inulin Improves Diet-Induced Hepatic Steatosis and Increases Intestinal Akkermansia Genus Level
Source: Int J Mol Sci. 2022 Jan 17;23(2):991. doi: 10.3390/ijms23020991 (PMC8782000; doi:10.3390/ijms23020991)
Supplement: Supplementary file 1 [file ijms-23-00991-s001.zip › Supp.Fig.Caption.pdf]

Figure S1. **A**) Rarefaction curves of the sequencing depth corresponding to the four mice groups either, at the beginning (I) or the end (F) of the experimental procedures. **B**) The alpha diversity of the four mice groups is presented with the corresponding significance for the initial or final relative abundance for each mice group. C-C, chow-cellulose; C-I, chow-inulin; H-C, fat-enriched cellulose; H-I, fat-enriched inulin. # $p<0.05$, one-way Anova, Tukey´s test.

Figure S2. **A**) The relative abundance at the Species level is presented for each mice group for the initial (I) or final (F) period of supplementation. **B**) Linear Discriminant Analysis effect size (LEfSE) of the features with statistical significance change between the initial and final periods for the H-I group.

Figure S3. **A**) Pearson´s correlation of liver damage markers or triglycerides and cholesterol with *A. muciniphila*, in the H-I mice group; the numbers inside squares represents the R-values and the color indicates the correlation direction. **B**) Heatmap showing Pearson´s correlation for multiple ASVs and serum metabolites in the H-I mice group at the end of the experimental procedure. See main text for details.

Figure S4. Inulin supplementation-derived enriched metabolic pathways. Metagenome inference indicates that propionate (Prop._metab.), secondary metabolites (Biosyn./biodeg./sec./metab), fatty acid (Fatty_acid_metabolism), biotin (Biotin_metabolism) and Gluthatione (Glutha-tione_metabolism) are enriched after inulin treatment in the H-I group. Plot indicates the enriched metabolic pathways before (I) or after (F) the eight-week period of inulin supplementation. ** $p<0.01$ (I vs. F) for each metabolic pathway.
