# Supplementary figures and images for: Inulin Improves Diet-Induced Hepatic Steatosis and Increases Intestinal Akkermansia Genus Level
Source: Int J Mol Sci. 2022 Jan 17;23(2):991. doi: 10.3390/ijms23020991 (PMC8782000; doi:10.3390/ijms23020991)

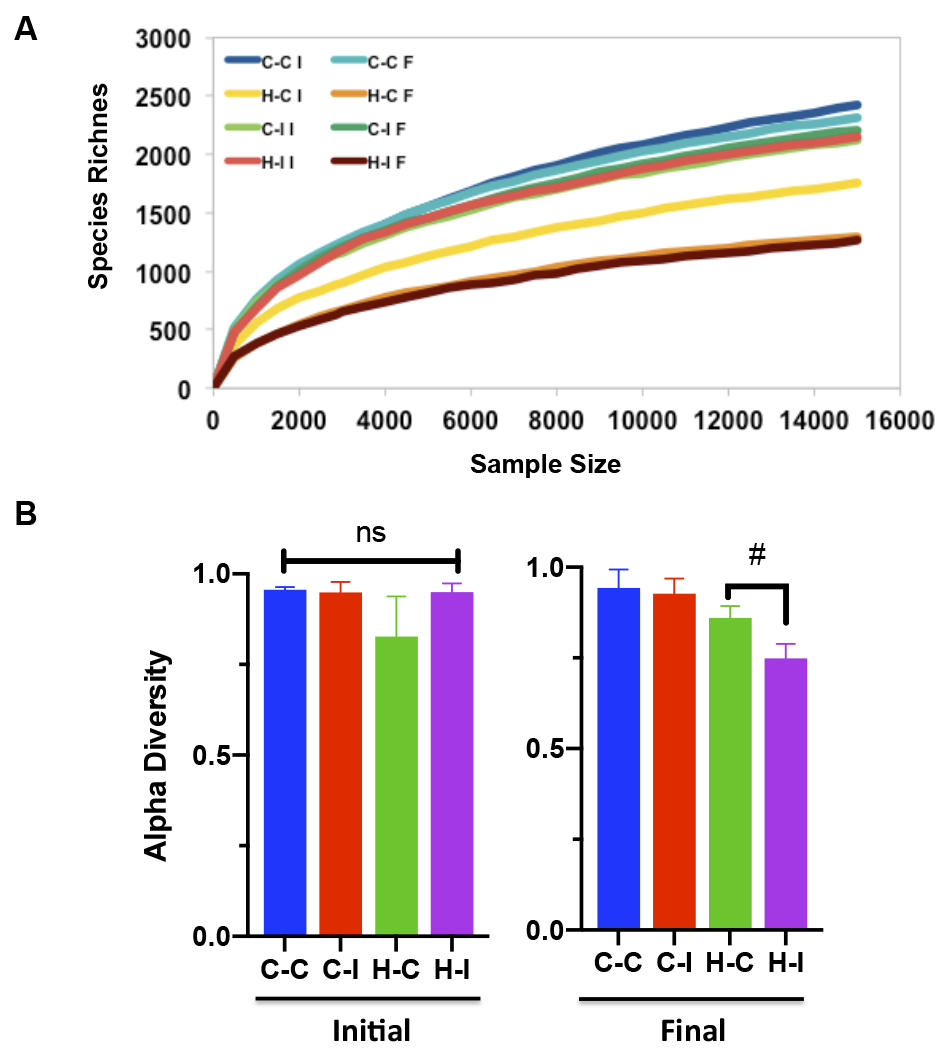

Supplement: Supplementary file 1 [file ijms-23-00991-s001.zip › Supp.Figure S1.tif]

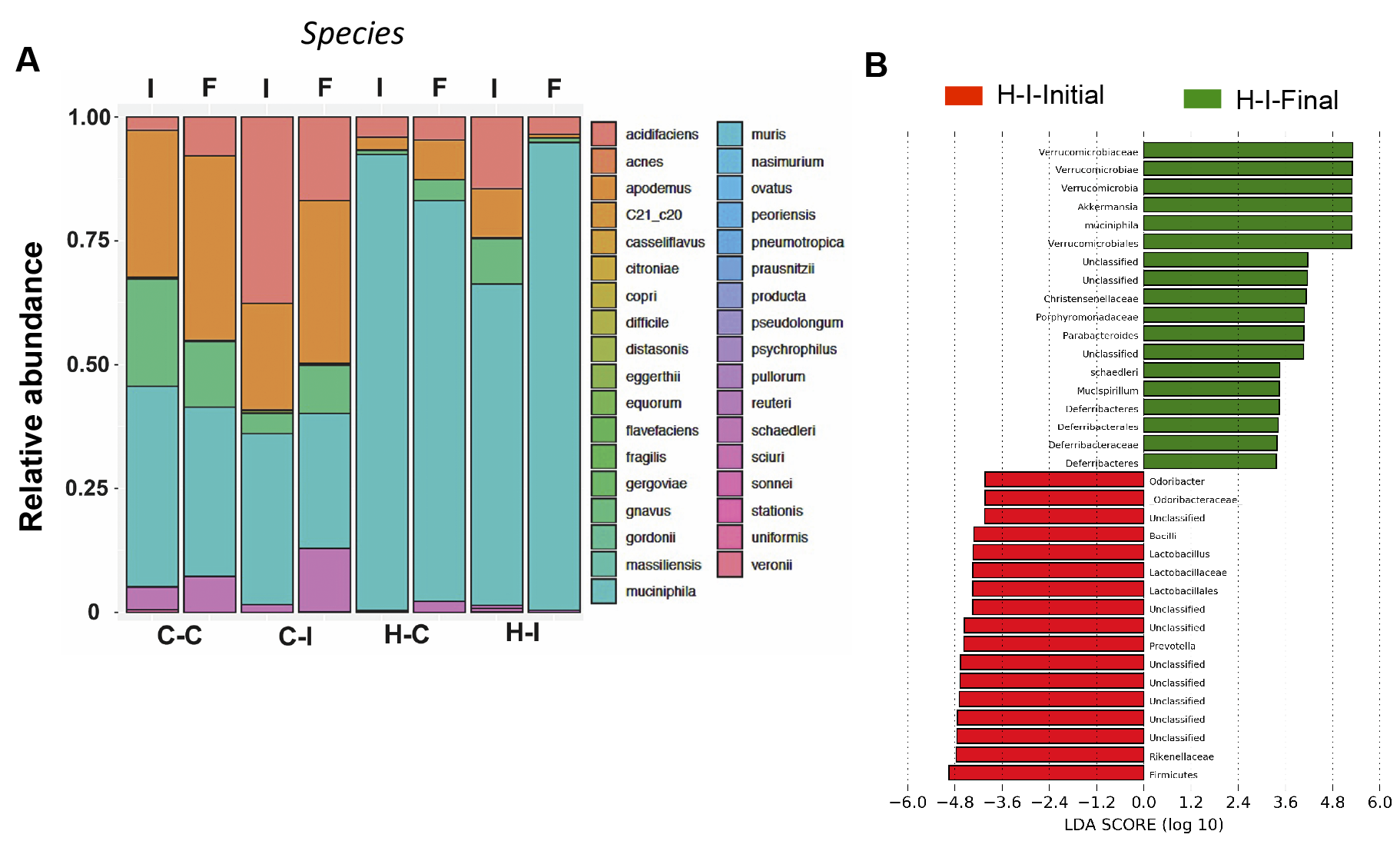

Supplement: Supplementary file 1 [file ijms-23-00991-s001.zip › Supp.Figure S2.tif]

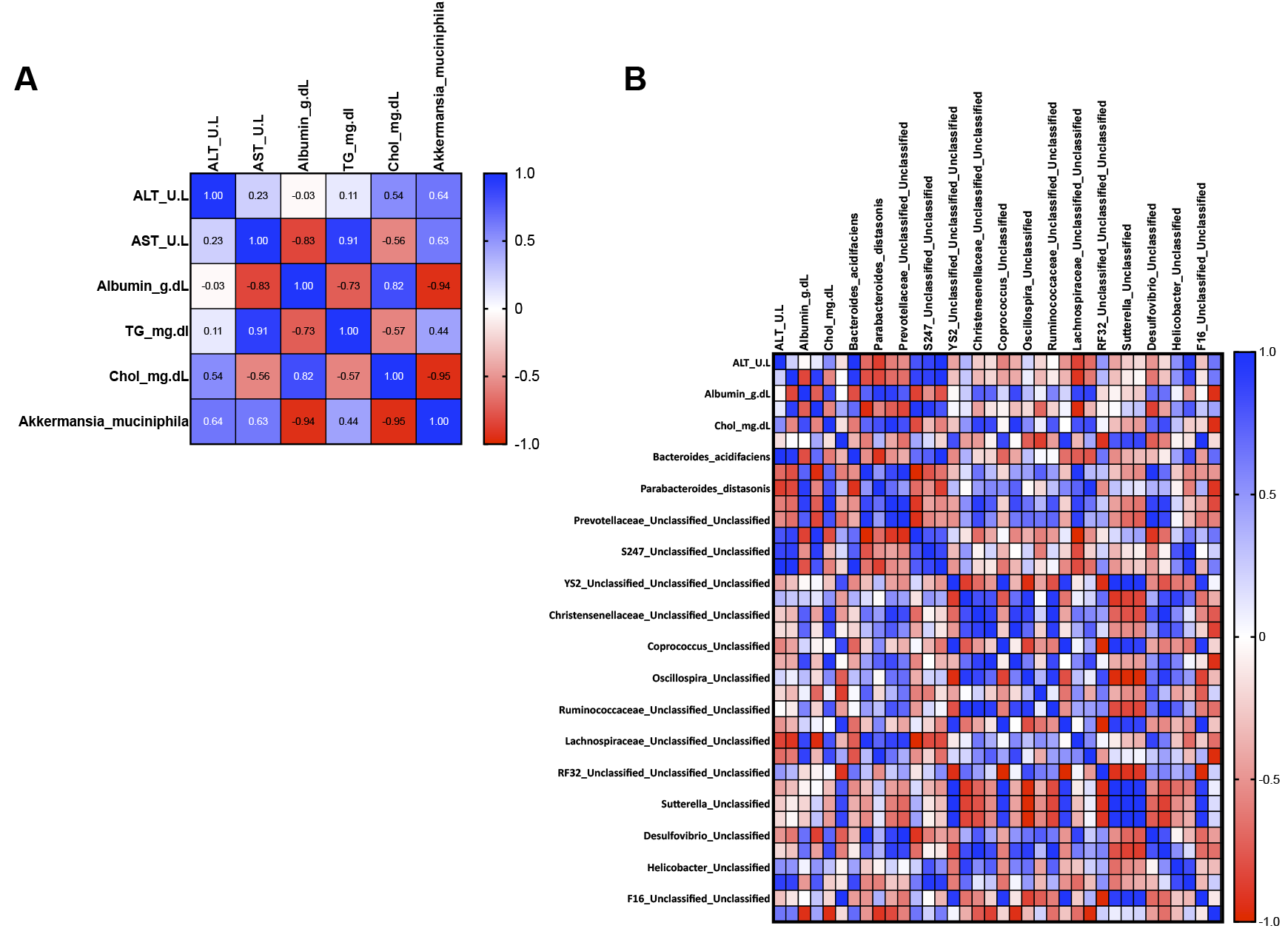

Supplement: Supplementary file 1 [file ijms-23-00991-s001.zip › Supp.Figure S3.tif]

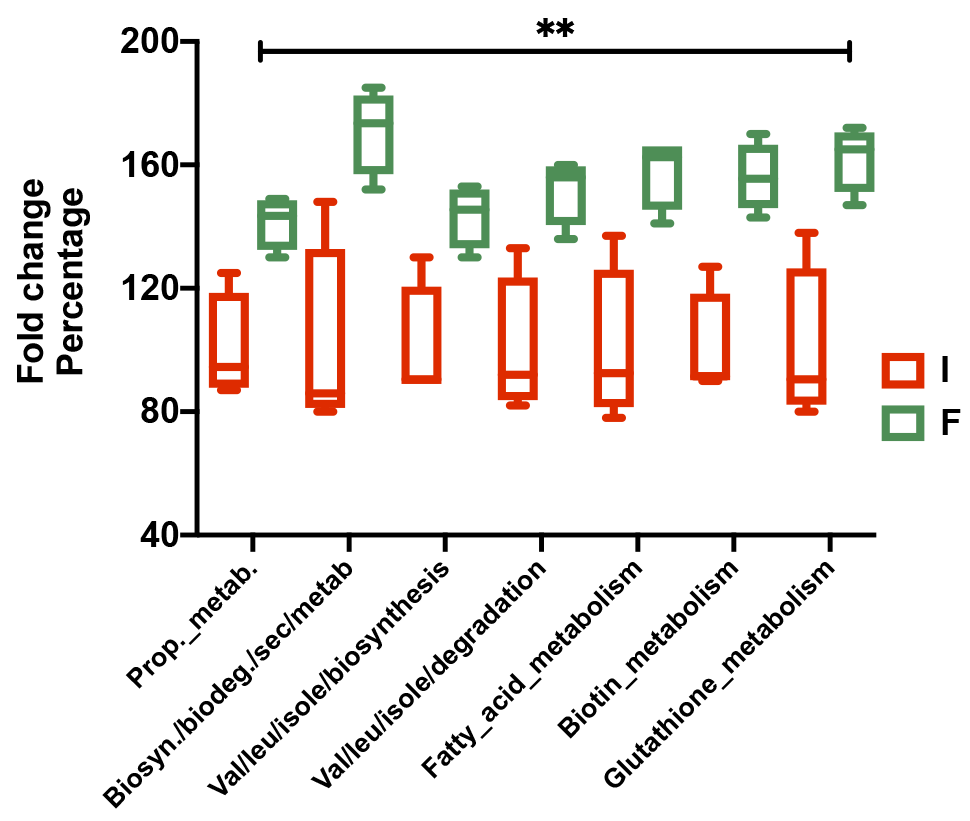

Supplement: Supplementary file 1 [file ijms-23-00991-s001.zip › Supp.Figure S4.tif]
